# Comparative analysis of prognostic assessment in hospitalized heart failure patients: a comprehensive evaluation of KDIGO and WRF classifications

**DOI:** 10.3389/fcvm.2025.1447994

**Published:** 2025-04-23

**Authors:** Chien-Hao Su, Pei-Chun Fan, Ya-Lien Cheng, Pao-Chu Wu, Chao-Yu Chen, Cheng-Chia Lee, Yung-Chang Chen, Victor Chien-Chia Wu, Pao-Hsien Chu, Chih-Hsiang Chang

**Affiliations:** ^1^ School of Pharmacy, Kaohsiung Medical University, Kaohsiung, Taiwan; ^2^ Department of Pharmacy, Chang Gung Memorial Hospital, Kaohsiung, Taiwan; ^3^ Department of Nephrology, Kidney Research Center, Linkou Medical Center, Taoyuan, Taiwan; ^4^ Graduate Institute of Clinical Medicine Science, College of Medicine, Chang Gung University, Taoyuan, Taiwan; ^5^ Department of Cardiology, Chang Gung Memorial Hospital, Taoyuan, Taiwan

**Keywords:** acute decompensated heart failure, acute kidney injury, worsening renal function, mortality, major adverse kidney effects

## Abstract

**Introduction:**

The definition of acute kidney dysfunction in patients with acute decompensated heart failure (ADHF) remains unclear. This study aimed to compare two sets of criteria for acute kidney injury (AKI), namely, the kidney disease: improving global outcomes (KDIGO) and worsening renal function (WRF) classification, in hospitalized patients with ADHF.

**Methods:**

We utilized a multi-institutional database with 17,684 cases of hospitalizations for HF. AKI was defined using KDIGO, WRF-serum creatinine (Scr), and WRF-estimated glomerular filtration rate (eGFR) criteria. The study compared the performance of these criteria in predicting in-hospital mortality and employed logistic regression to assess associations with mortality, HF hospitalization, and major adverse kidney effects (MAKE). A sensitivity analysis was conducted to compare the modified KDIGO (mKDIGO) with the traditional AKI criteria.

**Results:**

The incidences of ADHF according to the KDIGO, WRF-Scr, and WRF-eGFR criteria were 28.6%, 29.9%, and 29.9%, respectively. KDIGO exhibited higher discriminatory power compared with WRF-Scr and WRF-eGFR for in-hospital mortality[area under the curve (AUC):73.6% vs. 71.6% vs. 71.2%]. On all definitions, ADHF was predicted to have an increase in mortality and MAKE, with mortality increasing stepwise with AKI severity. A sensitivity analysis revealed mKDIGO to be more accurate than WRF criteria for identifying in-hospital mortality and recognizing AKI early.

**Conclusions:**

In hospitalized patients with ADHF, KDIGO is a more effective predictive tool for in-hospital mortality compared with WRF classification. Integrating a newer severity-staging classification into WRF criteria may enhance their predictive association with poor prognosis and enable early intervention.

## Introduction

Acute kidney injury (AKI) is a prevalent condition that is associated with increases in incident chronic kidney disease (CKD) risk, CKD progression, cardiovascular events, and re-hospitalization or mortality rates, whether all-cause or related to specific causes (1, 2). Unlike early AKI recovery, progression to acute kidney disease (AKD) significantly elevates the risk of 1-year mortality and major adverse kidney events (MAKE) (3, 4). Factors contributing to AKD risk include increased AKI severity, existing cancer or chronic heart failure (HF), and recent use of loop diuretics (3, 4). In patients with acute decompensated HF (ADHF), worsening renal function (WRF) is commonly mentioned as changes in kidney function (5). However, definitions of WRF differ considerably in serum creatinine changes and glomerular filtration rate. Reports indicate that 23% of patients with ADHF develop WRF during hospitalization, which increases the risk of death and subsequent hospital admissions (6, 7). Nevertheless, outcomes for patients experiencing transient or persistent WRF are conflicting, partly due to inconsistencies in WRF diagnostic criteria (8–11). Although the severity of WRF is associated with increased mortality, a universally accepted WRF staging algorithm is lacking (6, 12). Moreover, permissive creatinine elevation has been observed in patients with ADHF undergoing decongestive therapy.

AKI has been characterized as a rapid decline in kidney function within 48 h, indicated by an absolute increase in serum creatinine of ≥0.3 mg/dl (≥26.4 μmol/L) or a percentage increase of ≥50% (1.5-fold from baseline) within 7 days. Although nephrologists widely endorse the definitions and staging system of AKI derived from the kidney disease: improving global outcomes (KDIGO), acute kidney injury network (AKIN), and risk, injury, failure, loss of kidney function, and end-stage kidney disease (RIFLE) classifications, a consensus on criteria for classifying AKI and WRF is absent among cardiologists (13). Roy et al. discovered that adopting newer AKI classification marginally enhances the prediction of composite adverse outcomes at 30 days and 1 year over traditional WRF definitions (14). Moreover, the risks of mortality and HF readmissions escalate exponentially with AKI severity (14).

Numerous investigations have revealed that patients of African descent with HF or AKI exhibit enhanced survival rates compared with their counterparts of European descent (15, 16). However, studies systematically comparing various definitions and severity classifications of WRF against KDIGO AKI criteria for prognostic prediction in HF, particularly within patients of Asian descent, remain limited. In addition, although there was no classification of the severity of WRF, we established stratification for the fold changes of serum Cr and GFR to better understand the difference between these definitions of renal dysfunction in patients with ADHF.

## Materials and methods

### Ethics

The Institutional Review Board of Chang Gung Memorial Hospital approved this study (Institutional Review Board number: 202000915B0). The requirement for individual consent was waived due to the anonymized nature of the Chang Gung research database (CGRD).

### Data source

This retrospective cohort study was conducted using data extracted from the CGRD. The Chang Gung Medical Foundation is the largest medical system in Taiwan and operates seven hospitals across the country. The CGRD is a comprehensive multi-institutional electronic medical record database that offers extensive clinical information, including detailed laboratory results and hemodynamic records, offering broader coverage compared with standard claims databases. Its high overall and disease-specific coverage of the Taiwanese population is well-documented (17, 18). Disease identification in this study was based on employed the international classification of diseases (ICD), ninth revision, clinical modification (ICD-9-CM) diagnostic codes for records before 2015, and ICD, tenth revision (ICD-10), clinical modification for records after 2016.

### Study population

**Figure 1:**
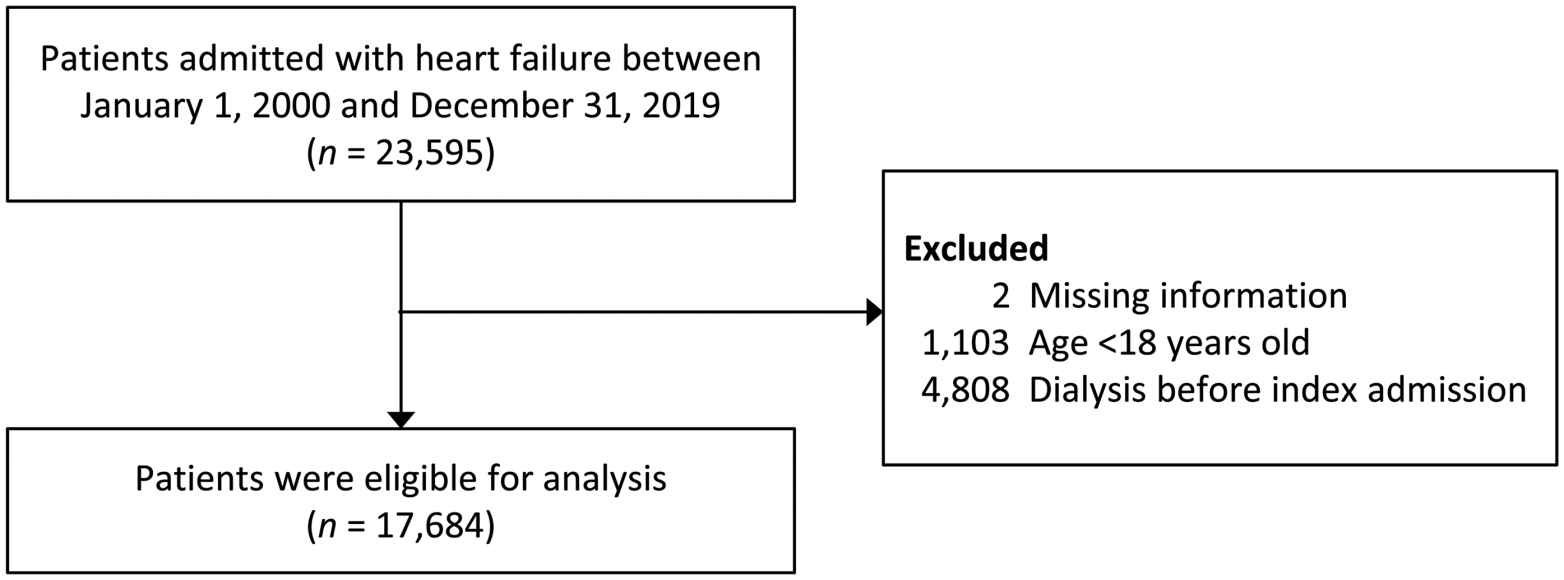
Flowchart of patient inclusion. SCr, serum creatinine; AKI, acute kidney injury.

The study identified individuals admitted with HF using ICD-9-CM code 428 and ICD-10 code I50 as discharge diagnoses, accompanied by at least one heart failure treatment during hospitalization (including diuretics, nitrites, or inotropic agents) at any time from January 1, 2000, to December 31, 2019, in the CGRD. Patients with adequate data for determining AKI were selected [i.e., data on baseline and subsequent serum creatinine (SCr) examinations during hospitalization]. For those with multiple HF hospitalizations, the first admission was designated as the index admission. Exclusions included individuals under 18 years and those who had undergone dialysis before their initial index admission (Figure 1).

### AKI and WRF definitions

The study targeted patients with serial renal function measurements throughout the study period. Determination of AKI involved continuously comparing a patient's creatinine levels against their lowest creatinine level over the preceding 7 days, consistent with the KDIGO AKI criteria (18). The initial occurrence of AKI during the index admission was considered the index date. WRF was defined as either a SCr increase to ≥0.3 mg/dl or an estimated glomerular filtration rate (eGFR) decline of ≥20% from the baseline creatinine during the index HF admission. WRF was categorized into creatinine-based (WRF-Scr) and eGFR-based (WRF-eGFR) groups (5).

To establish baseline creatinine, the study considered the nearest creatinine measurement within 3 months before the index admission, the first creatinine level during that admission, or the lowest level if no measurement was available within 3 months of the index admission. AKI occurrence was determined if any of the three criteria (KDIGO, WRF-Scr, WRF-GFR) were met. Since there was no definition of AKI severity in the definition of WRF, we want to explore not only the comparison between WRF and KDIGO but also AKI staging. Therefore, AKI severity was staged based on the fold change in creatinine or the percentage of eGFR reduction from baseline (refer to Table 1). In a sensitivity analysis, this study re-evaluated renal function trajectories, categorizing cases with a Scr increase to ≥4 mg/dl and a rise of 0.3 mg/dl within 48 h as stage I, thus redefining the modified KDIGO (mKDIGO) criteria.

**Table 1:** Severity of AKI classification according to three definition in current study.

Classification	Criteria	Definition	Time to AKI occurs
KDIGO			
Stage 1	(1)	≥1.5–1.9 times of baseline	≤7 days
	(2)	< 4.0 mg/dl with 0.3 mg/dl increase but <1.5 times baseline	≤48 h
Stage 2	(1)	≥2–2.9 times of baseline	≤7 days
Stage 3	(1)	≥3 times of baseline	≤7 days
	(2)	≥4.0 mg/dl with 0.3 mg/dl increase	≤48 h
	(D)	AKI requiring dialysis	
Baseline refer to the lowest serum creatinine fulfilled with criteria 1			
Modified KDIGO (mKDIGO)			
Stage 1	(1)	≥1.5–1.9 times of baseline	≤7 days
	(2)	0.3 mg/dl increase but <1.5 times of baseline	≤48 h
Stage 2	(1)	≥2–2.9 times of baseline	≤7 days
Stage 3	(1)	≥3 times of baseline	≤7 days
	(D)	AKI requiring dialysis	
Baseline refer to the lowest serum creatinine fulfilled with criteria 1			
WRF-Scr			
Stage 1	(1)	≥1.5–1.9 times of baseline	Dynamically occurs at any time during admission.
	(2)	< 1.5 times of baseline but increase in ≥0.3 mg/dl from baseline	Dynamically occurs at any time during admission.
Stage 2	(1)	≥2–2.9 times of baseline	Dynamically occurs at any time during admission.
Stage 3	(1)	≥3 times of baseline	Dynamically occurs at any time during admission.
	(D)	WRF requiring dialysis	
Baseline refers to the nearest serum creatinine checked before admission or the first creatinine checked at admission			
WRF-eGFR			
Stage 1		Decrease in eGFR of ≥20–49%	Dynamically occurs at any time during admission.
Stage 2		Decrease in eGFR of ≥50–74%	Dynamically occurs at any time during admission.
Stage 3		Decrease in eGFR of ≥75%	Dynamically occurs at any time during admission.
Stage 3-D		WRF requiring dialysis	

### Measurement of covariates

The study collected data on patient characteristics including age, gender, body mass index, baseline renal function (i.e., serum creatinine, eGFR and CKD stage), comorbidities, and left ventricular ejection fraction during the index admission. Hemodynamic parameters, including systolic blood pressure, diastolic blood pressure, and heart rate were documented upon arrival at the emergency room or on the day of admission. The initial set of laboratory results during the index admission, comprising measurements such as hemoglobin (Hb), blood urea nitrogen, serum creatinine, albumin, sodium, potassium, proteinuria, and B-type natriuretic peptide (BNP) levels, was also included. Medication prescriptions within the 3 months preceding the index admission were extracted to provide a comprehensive overview.

### Outcome definition

This study primarily aimed to assess and compare the predictive efficacy of various AKI diagnostic and staging classifications concerning in-hospital mortality, as well as all-cause death and HF hospitalization (HHF) at discharge, 90 days, 365 days, and 1 year post-discharge MAKE. MAKE was defined as the occurrence of end-stage renal disease requiring long-term renal replacement therapy or the development of new-onset CKD, determined by an eGFR <60 ml/min per 1.73 m^2^ according to the Modification of Diet in Renal Disease equation. The secondary goal was to analyze the timing of incident AKI events after applying different AKI definitions in patients hospitalized for HF.

### Statistical analysis

All statistical analyses were conducted using SAS 9.4 for Windows (SAS Institute, Cary, NC, USA). The significance level was set at 0.05 (two-tailed). To compare baseline characteristics between patients with and without AKI, an independent sample *t*-test was employed for continuous variables, a Mann–Whitney *U*-test for skewed continuous variables (e.g., troponin-I and lactic acid), and a Chi-square test for categorical variables. The discriminative ability of the criteria in predicting patient's prognosis was evaluated by calculating the area under the curve (AUC) of the receiver operating characteristic curve. The AUCs of the different criteria were compared using DeLong's nonparametric approach. Multiple logistic regression analysis was applied to assess the association of each KDIGO AKI, WRF-Scr, and WRF-eGFR category and AKI severity with the primary outcome, with adjustments for age, gender, eGFR at admission, diabetes mellitus, dyslipidemia, hypertension, myocardial infarction, and atrial fibrillation.

## Results

### Patient inclusion

The patient inclusion process is illustrated in Figure 1. Initially, 23,595 patients admitted for HF with adequate data for AKI assessment were identified between January 1, 2000 and December 31, 2019. After applying exclusion criteria, 17,684 patients qualified for analysis, with 56.1% being male. The mean age was 71.2 ± 14.6 years, and the average hospital stay was 16.7 ± 13.1 days. Among these patients, 6,592 (37.3%) developed AKI per any of the three criteria. Of these, 2,513 (14.2%) died at discharge.

### Patient characteristics

Patients who developed AKI had a lower mean eGFR (52.4 ± 42.1 vs. 57.0 ± 31.0 ml/min, *p* < 0.001), higher prevalence of diabetes mellitus and atrial fibrillation, and were more likely exposed to nephrotoxic agents such as nonsteroidal anti-inflammatory drugs and diuretics compared with those without AKI. Additional demographic characteristics are detailed in Table 2.

**Table 2:** Baseline patient characteristics according to the status of AKI/WRF based on any of the three criteria.

Variable	Available number	AKI (*n* = 6,592)	Non-AKI (*n* = 11,092)	*P* value
Baseline demographics				
Age, year	17,684	71.2 ± 14.6	71.1 ± 14.9	0.766
Male	17,684	3,612 (54.8)	6,316 (56.9)	0.005
Body mass index, kg/m^2^	14,704	24.6 ± 5.1	24.9 ± 5.3	<0.001
Renal function at admission				
Creatinine, mg/dl	17,684	2.3 ± 2.3	1.6 ± 1.1	<0.001
eGFR, ml/min/1.73 m^2^	17,684	52.4 ± 42.1	57.0 ± 31.0	<0.001
eGFR stages	17,684			<0.001
G1 (≥90)		912 (13.8)	1,306 (11.8)	
G2 (60–89)		1,383 (21.0)	3,271 (29.5)	
G3a (45–59)		1,019 (15.5)	2,348 (21.2)	
G3b (30–44)		1,075 (16.3)	2,100 (18.9)	
G4 (15–29)		1,140 (17.3)	1,523 (13.7)	
G5 (<15)		1,063 (16.1)	544 (4.9)	
Comorbidity				
Diabetes mellitus	17,684	3,087 (46.8)	4,685 (42.2)	<0.001
Dyslipidemia	17,684	2,139 (32.4)	3,745 (33.8)	0.073
Hypertension	17,684	4,410 (66.9)	7,470 (67.3)	0.541
Myocardial infarction	17,684	386 (5.9)	709 (6.4)	0.152
Atrial fibrillation	17,684	1,801 (27.3)	3,425 (30.9)	<0.001
Heart function				
LVEF, %	5,571	62.9 ± 13.8	61.5 ± 14.4	<0.001
LVEF group	5,571			<0.001
<40% (reduced)		151 (7.6)	346 (9.7)	
40–54%		279 (14.0)	605 (16.9)	
≥55% (preserved)		1,560 (78.4)	2,630 (73.4)	
Vital sign				
SBP, mmHg	16,494	135.0 ± 29.7	133.5 ± 27.5	0.001
DBP, mmHg	16,489	76.1 ± 18.6	77.4 ± 18.3	<0.001
Heart rate, beat/min	16,494	92.0 ± 22.8	89.7 ± 22.6	<0.001
Baseline lab data				
Hemoglobin, g/dl	17,680	11.3 ± 2.6	12.1 ± 2.6	<0.001
Platelets, 1,000/ul	17,675	211.1 ± 98.1	213.5 ± 90.8	0.099
BUN, mg/dl	17,438	39.4 ± 29.9	29.4 ± 20.6	<0.001
Bicarbonate, mmol/L	8,916	22.6 ± 6.9	24.0 ± 6.3	<0.001
Sodium, mg/dl	17,665	137.1 ± 6.3	137.6 ± 5.6	<0.001
Potassium, mg/dl	17,673	4.1 ± 0.8	4.0 ± 0.7	<0.001
Albumin, mg/dl	14,153	3.2 ± 0.7	3.4 ± 0.6	<0.001
Proteinuria, mg/dl	13,972			<0.001
Negative (0–4)		1,560 (28.1)	3,453 (41.0)	
Trace (5–29)		742 (13.4)	1,180 (14.0)	
≥1+ (≥30)		3,243 (58.5)	3,794 (45.0)	
BNP, pg/ml	8,275	1,093 [501, 2,198]	808 [382, 1,549]	<0.001
NT-pro BNP, pg/ml	409	6,139 [2,608, 15,200]	3,047 [1,192, 8,438]	<0.001
Troponin-I, ng/ml	14,527	0.19 [0.05, 1.40]	0.08 [0.03, 0.48]	<0.001
Lactic acid, mg/dl	4,234	21.0 [12.6, 41.9]	18.2 [11.9, 30.1]	<0.001
pH	8,272	7.38 ± 0.12	7.40 ± 0.11	<0.001
Medication treatment				
ACEi/ARB	17,684	4,654 (70.6)	8,162 (73.6)	<0.001
Sacubitril/valsartan	17,684	53 (0.8)	97 (0.9)	0.621
Ivabradine	17,684	11 (0.2)	29 (0.3)	0.200
SGLT2i	17,684	74 (1.1)	172 (1.6)	0.019
MRA	17,684	1,919 (29.1)	3,295 (29.7)	0.401
Digoxin	17,684	1,609 (24.4)	2,388 (21.5)	<0.001
Calcium channel blocker	17,684	3,837 (58.2)	5,118 (46.1)	<0.001
Beta-blocker	17,684	4,477 (67.9)	7,150 (64.5)	<0.001
Loop-diuretics	17,684	6,032 (91.5)	9,244 (83.3)	<0.001
Vasodilators	17,684	1,493 (22.6)	1,225 (11.0)	<0.001
NSAIDs	17,684	2,617 (39.7)	3,542 (31.9)	<0.001

AKI, acute kidney injury; WRF, worsening renal function; CKD, chronic kidney disease; eGFR, estimated glomerular filtration rate; LVEF, left ventricular ejection fraction; SBP, systolic blood pressure; DBP, diastolic blood pressure; BUN, blood urea nitrogen; BNP, B-type natriuretic peptide; ACEi, angiotensin converting enzyme inhibitor; ARB, angiotensin receptor blocker; SGLT2i, sodium-glucose cotransporter 2 inhibitor; MRA, mineralocorticoid receptor antagonist NSAIDs, non-steroidal anti-inflammatory drugs;.

Data were presented as frequency (percentage), mean ± standard deviation or median [25th, 75th percentiles].

### Incidence of AKI using different definitions

AKI defined per the KDIGO classification was identified in 5,051 (28.6%) patients, with 51.8% classified as stage 1, 13.6% as stage 2, 14.3% as stage 3, and 20.3% requiring dialysis. AKI defined per the WRF-Scr criteria was identified in 5,286 (29.9%) patients, with 61.1% at stage 1, 12.7% at stage 2, 6.8% at stage 3, and 19.4% requiring dialysis. AKI defined per the WRF-eGFR criteria was identified in 5,284 (29.9%) patients, with 56.5% at stage 1, 18.9% at stage 2, 5.3% at stage 3, and 19.4% requiring dialysis. (Supplementary Table S1). Fewer patients had AKI according to the KDIGO criteria than the WRF criteria. Supplementary Tables S1,S2 provides a detailed cross-tabulation of different AKI criteria and interested outcome in the present study.

### In-hospital death and 3-month outcomes

AKI was associated with elevated in-hospital and 90-day mortality, with the risk increasing with AKI severity as defined by any criterion. This association persisted after adjusting for factors such as eGFR at admission, age, gender, and underlying conditions such as diabetes mellitus, dyslipidemia, hypertension, myocardial infarction, and atrial fibrillation (see Tables 3, 4).

**Table 3:** In-hospital death and outcomes during 3-month follow up after discharge under different AKI/WRF classifications.

Outcome/definition	AKI or WRF = yes		AKI or WRF = no		AUC, % (95% CI)	aOR (95% CI)^a^
	*n*	Event (%)	*n*	Event (%)		
In-hospital death						
KDIGO	5,051	1,734 (34.3)	12,633	779 (6.2)	73.6 (72.6–74.5)	8.56 (7.78–9.42)*
WRF SCr	5,286	1,682 (31.8)	12,398	831 (6.7)	71.6 (70.6–72.6)	6.92 (6.29–7.60)*
WRF GFR	5,284	1,664 (31.5)	12,400	849 (6.9)	71.2 (70.2–72.2)	6.61 (6.03–7.26)*
All-cause death						
KDIGO	3,317	357 (10.8)	11,854	917 (7.7)	53.4 (52.1–54.6)	1.53 (1.34–1.75)*
WRF SCr	3,604	395 (11.0)	11,567	879 (7.6)	54.0 (52.6–55.3)	1.52 (1.33–1.73)*
WRF GFR	3,620	371 (10.3)	11,551	903 (7.8)	52.9 (51.6–54.2)	1.44 (1.26–1.63)*
Heart failure hospitalization^b^						
KDIGO	3,317	179 (5.4)	11,854	577 (4.9)	51.0 (49.4–52.5)	1.02 (0.85–1.21)
WRF SCr	3,604	213 (5.9)	11,567	543 (4.7)	52.3 (50.7–54.0)	1.14 (0.97–1.35)
WRF GFR	3,620	179 (4.9)	11,551	577 (5.0)	50.1 (48.5–51.7)	0.97 (0.82–1.15)

AKI, acute kidney injury; WRF, worsening renal function; AUC, area under the curve; aOR, adjusted odds ratio; CI, confidence interval; KDIGO, kidney disease: improving global outcomes; SCr, serum creatinine; GFR, glomerular filtration rate.

^a^ Adjusted for eGFR at admission, age, gender, underlying diabetes mellitus, dyslipidemia, hypertension, myocardial infarction and atrial fibrillation;.

^b^ Patients who survived to discharge were included in the analysis.

*Represents significants difference.

**Table 4:** In-hospital death and outcomes during 3-month follow up after discharge by different AKI stages under different classifications.

Outcome/stage	KDIGO			WRF SCr			WRF GFR		
	*n*	Event (%)	aOR (95% CI)^a^	*n*	Event (%)	aOR (95% CI)^a^	*n*	Event (%)	aOR (95% CI)^a^
In-hospital death									
0	12,633	779 (6.2)	Reference	12,398	831 (6.7)	Reference	12,400	849 (6.9)	Reference
1	2,615	707 (27.0)	5.79 (5.16–6.50)*	3,232	672 (20.8)	3.76 (3.36–4.22)*	2,983	575 (19.3)	3.37 (3.00–3.79)*
2	687	316 (46.0)	13.08 (11.01–15.53)*	671	331 (49.3)	13.89 (11.69–16.50)*	997	452 (45.3)	12.05 (10.39–13.97)*
3	724	256 (35.4)	10.31 (8.63–12.32)*	358	224 (62.6)	25.43 (20.12–32.13)*	279	182 (65.2)	30.54 (23.36–39.92)*
Dialysis	1,025	455 (44.4)	16.02 (13.70–18.73)*	1,025	455 (44.4)	13.67 (11.70–15.98)*	1,025	455 (44.4)	12.15 (10.42–14.17)*
All-cause death^b^									
0	11,854	917 (7.7)	Reference	11,567	879 (7.6)	Reference	11,551	903 (7.8)	Reference
1	1,908	202 (10.6)	1.48 (1.25–1.74)*	2,560	277 (10.8)	1.45 (1.26–1.68)*	2,408	236 (9.8)	1.35 (1.16–1.58)*
2	371	45 (12.1)	1.70 (1.23–2.36)*	340	45 (13.2)	1.84 (1.32–2.55)*	545	67 (12.3)	1.76 (1.34–2.30)*
3	468	55 (11.8)	1.70 (1.26–2.31)*	134	18 (13.4)	2.24 (1.33–3.75)*	97	13 (13.4)	2.32 (1.26–4.26)*
Dialysis	570	55 (9.7)	1.44 (1.07–1.96)*	570	55 (9.7)	1.45 (1.07–1.97)*	570	55 (9.7)	1.37 (1.01–1.85)*
HHF^b^									
0	11,854	577 (4.9)	Reference	11,567	543 (4.7)	Reference	11,551	577 (5.0)	Reference
1	1,908	109 (5.7)	1.18 (0.95–1.46)	2,560	169 (6.6)	1.30 (1.08–1.56)*	2,408	130 (5.4)	1.13 (0.92–1.37)
2	371	15 (4.0)	0.85 (0.50–1.43)	340	12 (3.5)	0.75 (0.42–1.34)	545	19 (3.5)	0.72 (0.45–1.15)
3	468	30 (6.4)	1.01 (0.68–1.50)	134	7 (5.2)	1.22 (0.57–2.64)	97	5 (5.2)	1.20 (0.49–2.99)
Dialysis	570	25 (4.4)	0.65 (0.42–0.99)*	570	25 (4.4)	0.69 (0.45–1.05)	570	25 (4.4)	0.64 (0.42–0.98)*

AKI, acute kidney injury; KDIGO, kidney disease: improving global outcomes; WRF, worsening renal function; SCr, serum creatinine; GFR, glomerular filtration rate; aOR, adjusted odds ratio; CI, confidence interval; HHF, heart failure hospitalization.

^a^ Adjusted for eGFR at admission, age, gender, underlying diabetes mellitus, dyslipidemia, hypertension, myocardial infarction and atrial fibrillation.

^b^ Patients who survived to discharge were included in the analysis.

*Represents significants difference.

The KDIGO-Scr AKI criteria had slightly higher AUC values for predicting in-hospital mortality compared with the WRF-Scr and WRF-eGFR criteria (AUC: 73.6% vs. 71.6% and 71.2%, respectively; *p* of DeLong's test <0.05; Table 3). The discriminative ability for in-hospital mortality slightly improved when AKI was identified using modified KDIGO (mKDIGO) criteria compared with KDIGO AKI criteria (AUC: 75.2% vs. 74.8%; *p* of DeLong's test <0.05; Supplementary Table S3).

Notably, no set of criteria predicted HFH, even at higher AKI severities or with adjustments for other variables (Tables 3, 4). However, the risk of 90-day HFH was significantly lower in patients with AKI requiring dialysis compared with those without AKI (Table 4).

### Outcomes during 1-year follow-Up

AKI, regardless of definition, was associated with an elevated risk of 1-year MAKE and death. However, this risk did not uniformly increase with increasing AKI severity (Supplementary Tables S4,S5). Similar to the 90-day findings, no association was observed between any of the AKI criteria studied and the risk of 1-year HFH. However, the initiation of dialysis following AKI significantly reduced the risk of 1-year HFH (Supplementary Table S5). Notably, both KDIGO and WRF criteria lacked meaningful discrimination ability in predicting the 1-year prognosis of patients.

### Outcomes of patients with WRF by the KDIGO status

Additional analysis was conducted to evaluate characteristics and outcomes for the patients who were included in WFR but not the KIDGO group. Specifically, the patients with WRF (either GFR or SCr definitions; *n* = 5,883) were classified into who fell outside of the KDIGO-AKI time window (*n* = 1,541) and who did not (*n* = 4,342). The characteristics of patients between these two groups were shown in the supplements (Supplementary Table S6). The results showed that patients with WRF who fell outside of the KDIGO-AKI time window had significantly lower risk of in-hospital death [10.6% vs. 36.5%, adjusted odds ratio [aOR] 0.19, 95% confidence interval [CI] 0.16–0.22] and 1-year MAKE (29.7% vs. 38.8%; aOR 0.68, 95% CI 0.50–0.94), compared to those who fell inside (Supplementary Table S7).

### Timing of AKI identification by various AKI definitions

**Figure 2:**
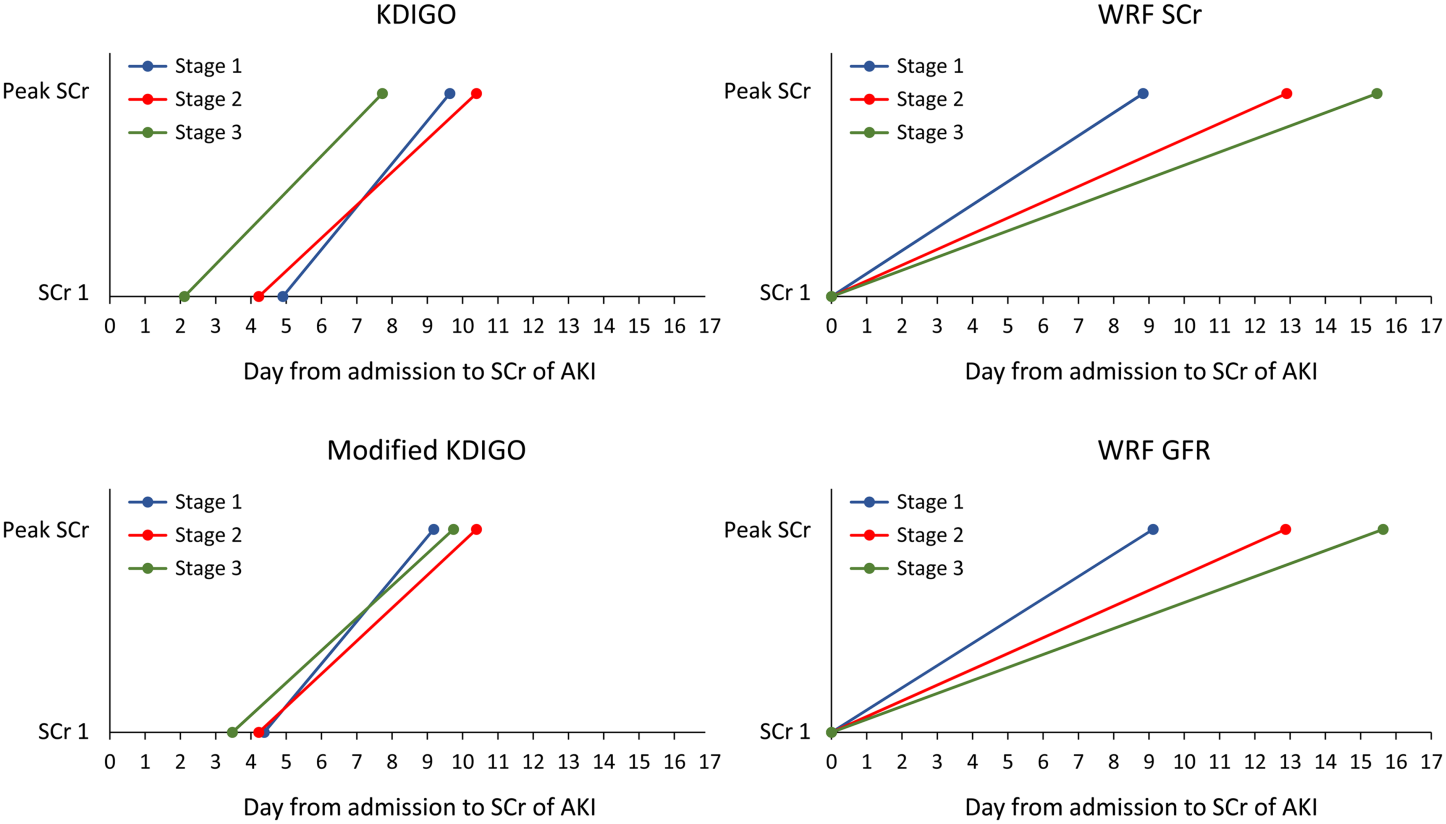
Timing of AKI identification by various AKI and WRF criteria. AKI, acute kidney injury; WRF, worsening renal function; KDIGO, Kidney Disease: Improving Global Outcomes; SCr, serum creatinine; GFR, glomerular filtration rate.

The KDIGO definition identified AKI cases earlier, with a mean of 9.4 ± 8.4 days post-index admission, compared with WRF-Scr or WRF-eGFR criteria (10.0 ± 10.4 days, *p* of paired *t* test <0.001). Under the KDIGO AKI definition, AKI stage 3 was the first to be identified, followed by stages 1 and 2 (Figure 2). The detection of AKI stages 1–3 was delayed when the WRF-Scr or WRF-eGFR criteria were applied. However, the mKDIGO AKI criteria offered a clinically reasonable and more straightforward timeframe for AKI detection compared with the traditional KDIGO and WRF criteria (Figure 2).

## Discussion

In the present study, the incidence of AKI was 37.3%, which is associated with a heightened risk of short-term mortality. This finding is consistent with relevant research on patients with acute HF (14, 19). Consistent with earlier findings, the KDIGO classification exhibited relatively higher discriminatory power for predicting in-hospital mortality among patients with ADHF compared with the WRF classifications (14). Our results align with those in the literature, indicating an exponential increase in short-term mortality risk (at discharge and 90 days) with increasing AKI severity, as defined by the criteria investigated in this study (6). This study is the first to propose a new classification of AKI severity for WRF criteria, offering superior short-term prognosis discrimination. Additionally, it contributes to the limited body of research evaluating the performance of KDIGO definitions against traditional WRF definitions in predicting in-hospital mortality in Asian populations with ADHF.

Individuals experiencing AKI were at an elevated risk of developing new or progressing CKD, ESKD, HF, and all-cause mortality, with a discernible risk gradient across AKI stages (20, 21). However, the prognostic significance of WRF in patients with ADHF remains controversial. Unstratified analyses indicate higher risks of post-discharge death or rehospitalization in patients with WRF (6, 22), and WRF severity is correlated with increased mortality (6). Studies that have disagreed with ours have suggested that WRF may not be inherently associated with adverse clinical outcomes in patients with HF. For instance, a prospective multicenter study indicated similar mortality and re-hospitalization rates for patients with ADHF with and without WRF (23), despite longer hospital stays in the WRF group. Another study found no association between WRF and cardiovascular mortality, major cardiovascular events, or the composite endpoint of four point-major adverse cardiovascular events (4P-MACE) in patients admitted for ADHF (24). Additionally, patients with WRF who achieved decongestion had superior prognoses than those without WRF or decongestion (25). These findings suggest that the traditional WRF classification may not effectively predict outcomes in patients with ADHF. Our study suggests that a newer WRF severity-staging classification could offer more accurate short-term risk predictions for death or MAKE in 1 year for hospitalized ADHF cohorts.

The KDIGO classification is similarly or better able to predict in-hospital mortality relative to the RIFLE or AKIN criteria in patients with critically illness, adults on extracorporeal membrane oxygenation (ECMO), and pediatric individuals undergoing heart surgery (26–29). In a previous study involving an ADHF cohort, the RIFLE and KDIGO definitions for predicting a composite of HF-related readmission, renal replacement therapy, and all-cause mortality at 30 days exhibited marginally superior AUC values compared with the WRF-Scr criteria (AUC 0.76 and 0.74 vs. 0.72, *p* = 0.02) (14). Additionally, a noticeable stepwise increase in the primary outcome occurred at higher AKI severities when the RIFLE, KDIGO, or AKIN definition was used (14). Our study corroborates these findings, observing that the KDIGO classification had a marginally higher discriminatory power than the WRF-Scr or eGFR definitions in predicting in-hospital mortality. The risks of in-hospital and 90-day mortality increased exponentially with the severity of AKI per the WRF criteria, which is a new staging system similar to KDIGO and RIFLE. However, although the risk of 1-year MAKE and death increased significantly after AKI, neither the KDIGO nor WRF criteria exhibited a concurrent increase with higher AKI severity categories. This discrepancy may be attributed to irregular creatinine measurement after HF discharge.

The effect of AKI on the risk of 90- and 365-day HFH did not differ significantly. However, patients with AKI requiring dialysis demonstrated a lower risk of HFH compared with those without AKI. A systematic review suggested that patients with WRF achieving decongestion had superior prognoses than those without WRF or decongestion (25). Another systematic review by Timóteo et al. reported an association between peritoneal dialysis and a significant reduction in hospitalization length, further emphasizing the potential benefits of fluid removal in providing decongestion in cases of heart failure and preventing HF re-hospitalization in ADHF cohorts with AKI requiring dialysis (30).

To mitigate the potential effects of mild creatinine fluctuations in HF patients with CKD on prognosis, we performed a sensitivity analysis. In this analysis, a subgroup of cases classified as AKI stage 3, with creatinine levels of ≥4 and a 0.3 mg/dl increase within 48 h, were reclassified to AKI stage 1. This analysis indicated that both the traditional and modified KDIGO criteria could identify AKI earlier than WRF criteria, potentially aiding in the prevention of CKD progression (see Supplementary Figure 1). Moreover, the mKDIGO criteria had better discriminatory power for in-hospital mortality relative to the traditional KDIGO criteria. This suggests that the modified criteria not only offer a timelier diagnosis of AKI but also enhance the predictive accuracy for short-term outcomes in patients with HF.

## Strengths and limitations

This study has several limitations. First, the retrospective observational cohort design precludes causal inference and carries a risk of selection bias.

Second, the limited number of biomarkers, such as troponin-I, NT-pro-BNP, and hs-CRP used as a marker of decongestion, only allowed us to control for a limited set of confounders in the predictive model. Nevertheless, employing a newer severity-staging classification for WRF offers enhanced risk estimation and provides a consistent framework akin to the widely accepted KDIGO AKI criteria. Third, based on the study design, we cannot define and detect the pseudo-WRF in this study. Pseudo-WRF is a condition with serum Cr change without intrinsic kidney injury, usually caused by hemodynamic or fluid changes. Fourth, we lacked data on urine output and fluid status, which are crucial for assessing renal function and fluid balance. Lastly, the findings may have been affected by ascertainment bias. Participants who experienced AKI episodes might have been less likely to undergo post-discharge renal function follow-up compared with those without AKI, which could potentially lead to an underestimation of the incidence of MAKE in patients with identified AKI episodes.

Nonetheless, one strength of this study is its large and diverse multi-institutional sample in Asian patients with ADHF. Furthermore, the study demonstrates the practical challenges of timely AKI detection in real-world clinical settings, particularly in the absence of an electronic alert system. Our findings underscore the efficacy of the KDIGO AKI criteria, based on short-term creatinine fluctuations, for early AKI identification and improved prognosis in patients with ADHF. This presents a pivotal opportunity for early intervention. Nonetheless, additional research is warranted to elucidate the effect of changes in decongestion biomarkers on various AKI criteria for prognosis prediction in this patient population.

## Conclusions

Among patients admitted for HF, the KDIGO classification demonstrated superior predictive ability for in-hospital mortality and earlier AKI detection compared with the WRF definition. Given the benefits of improved identification of high-risk populations, researchers and clinicians should work toward formulating a new severity-staging classification for WRF criteria. Additionally, regular post-discharge creatinine monitoring is essential to enhance renal prognosis prediction and inform the use of cardiovascular or renal protective medications.

## Data Availability

The datasets generated and analyzed during the current study are not publicly available due to the policy and regulation of the Institutional Review Board of Chang Gung Memorial Hospital. Requests to access the data should be directed to the corresponding author.
