# Supplementary material for: Comparative analysis of prognostic assessment in hospitalized heart failure patients: a comprehensive evaluation of KDIGO and WRF classifications
Source: Front Cardiovasc Med. 2025 Apr 23;12:1447994. doi: 10.3389/fcvm.2025.1447994 (PMC12055856; doi:10.3389/fcvm.2025.1447994)
Supplement: Supplementary file 1 [file Datasheet1.pdf]

Supplementary Table 1. Baseline patient characteristics according to the status of AKI or WRF

		KIDGO definition			WRF SCr definition			WRF GFR definition		
Variable	Available number	AKI (*n* = 5,051)	Non-AKI (*n* = 12,633)	*P*	AKI (*n* = 5,286)	Non-AKI (*n* = 12,398)	*P*	AKI (*n* = 5,284)	Non-AKI (*n* = 12,400)	*P*
Baseline demographics										
Age, year	17,684	70.7 ± 14.7	71.3 ± 14.9	0.036	71.5 ± 14.4	71.0 ± 15.0	0.036	71.1 ± 14.7	71.1 ± 14.9	0.907
Male	17,684	2,824 (55.9)	7,104 (56.2)	0.695	2,972 (56.2)	6,956 (56.1)	0.885	2,857 (54.1)	7,071 (57.0)	<0.001
Body mass index, kg/m^2	14,704	24.5 ± 5.0	24.9 ± 5.3	<0.001	24.7 ± 5.1	24.8 ± 5.3	0.226	24.6 ± 5.1	24.9 ± 5.3	0.017
Baseline of AKI judgment										
Creatinine, mg/dL	17,684	1.4 [0.9, 2.8]	1.3 [0.9, 1.8]	<0.001	1.6 [1.1, 2.9]	1.2 [0.9, 1.8]	<0.001	1.3 [0.9, 2.3]	1.3 [1.0, 1.9]	0.067
eGFR, mL/min/1.73m^2	17,684	47.5 [20.4, 78.5]	54.2 [35.3, 75.1]	<0.001	40.5 [19.8, 64.2]	54.9 [35.9, 75.5]	<0.001	50.1 [26.6, 76.2]	51.6 [32.3, 72.2]	0.006
eGFR stages	17,684			<0.001			<0.001			<0.001
G1 (≥90)		958 (19.0)	1,659 (13.1)		524 (9.9)	1,694 (13.7)		834 (15.8)	1,384 (11.2)	
G2 (60-89)		964 (19.1)	3,679 (29.1)		995 (18.8)	3,659 (29.5)		1,240 (23.5)	3,414 (27.5)	
G3a (45-59)		731 (14.5)	2,563 (20.3)		849 (16.1)	2,518 (20.3)		874 (16.5)	2,493 (20.1)	
G3b (30-44)		706 (14.0)	2,339 (18.5)		926 (17.5)	2,249 (18.1)		818 (15.5)	2,357 (19.0)	
G4 (15-29)		711 (14.1)	1,808 (14.3)		1,016 (19.2)	1,647 (13.3)		806 (15.3)	1,857 (15.0)	
G5 (<15)		981 (19.4)	585 (4.6)		976 (18.5)	631 (5.1)		712 (13.5)	895 (7.2)	
AKI Stage	17,684			NA			NA			NA
0		0 (0.0)	12,633 (100)		0 (0.0)	12,398 (100)		0 (0.0)	12,400 (100)	
1		2,615 (51.8)	-		3,232 (61.1)	-		2,983 (56.5)	-	
2		687 (13.6)	-		671 (12.7)	-		997 (18.9)	-	

Variable	Available number	KIDGO definition AKI (*n* = 5,051)	KIDGO definition Non-AKI (*n* = 12,633)	*P*	WRF SCr definition AKI (*n* = 5,286)	WRF SCr definition Non-AKI (*n* = 12,398)	*P*	WRF GFR definition AKI (*n* = 5,284)	WRF GFR definition Non-AKI (*n* = 12,400)	*P*
3		724 (14.3)	-		358 (6.8)	-		279 (5.3)	-	
Dialysis		1,025 (20.3)	-		1,025 (19.4)	-		1,025 (19.4)	-	
Underlying diseases										
Diabetes mellitus	17,684	2,354 (46.6)	5,418 (42.9)	<0.001	2,558 (48.4)	5,214 (42.1)	<0.001	2,423 (45.9)	5,349 (43.1)	0.001
Dyslipidemia	17,684	1,582 (31.3)	4,302 (34.1)	<0.001	1,729 (32.7)	4,155 (33.5)	0.299	1,671 (31.6)	4,213 (34.0)	0.002
Hypertension	17,684	3,337 (66.1)	8,543 (67.6)	0.046	3,606 (68.2)	8,274 (66.7)	0.055	3,491 (66.1)	8,389 (67.7)	0.040
Myocardial infarction	17,684	285 (5.6)	810 (6.4)	0.055	317 (6.0)	778 (6.3)	0.482	298 (5.6)	797 (6.4)	0.047
Atrial fibrillation	17,684	1,359 (26.9)	3,867 (30.6)	<0.001	1,386 (26.2)	3,840 (31.0)	<0.001	1,465 (27.7)	3,761 (30.3)	0.001
Heart function										
LVEF, %	5,571	62.8 ± 14.0	61.7 ± 14.3	0.012	63.1 ± 13.8	61.6 ± 14.3	<0.001	63.0 ± 13.8	61.6 ± 14.3	0.001
LVEF group	5,571			0.017			<0.001			0.001
<40% (Reduced)		117 (7.8)	380 (9.4)		115 (7.4)	382 (9.5)		119 (7.6)	378 (9.5)	
40-54%		216 (14.3)	668 (16.4)		208 (13.4)	676 (16.8)		216 (13.7)	668 (16.7)	
≥55% (Preserved)		1,174 (77.9)	3,016 (74.2)		1,230 (79.2)	2,960 (73.7)		1,240 (78.7)	2,950 (73.8)	
Vital sign										
SBP, mmHg	16,494	133.6 ± 30.1	134.2 ± 27.6	0.222	136.9 ± 30.2	132.9 ± 27.5	<0.001	135.6 ± 29.6	133.4 ± 27.8	<0.001
DBP, mmHg	16,489	75.5 ± 18.7	77.5 ± 18.3	<0.001	76.7 ± 18.8	77.0 ± 18.3	0.346	76.3 ± 18.4	77.2 ± 18.4	0.003
Heart rate, beat/min	16,494	92.6 ± 23.1	89.7 ± 22.4	<0.001	91.9 ± 22.7	89.9 ± 22.6	<0.001	92.1 ± 22.5	89.9 ± 22.7	<0.001
Baseline lab data										
Hemoglobin, g/dL	17,680	11.2 ± 2.6	12.0 ± 2.6	<0.001	11.1 ± 2.6	12.1 ± 2.6	<0.001	11.3 ± 2.6	12.0 ± 2.6	<0.001

Variable	Available number	KIDGO definition AKI (*n* = 5,051)	KIDGO definition Non-AKI (*n* = 12,633)	KIDGO definition *P*	WRF SCr definition AKI (*n* = 5,286)	WRF SCr definition Non-AKI (*n* = 12,398)	WRF SCr definition *P*	WRF GFR definition AKI (*n* = 5,284)	WRF GFR definition Non-AKI (*n* = 12,400)	WRF GFR definition *P*
Platelets, 1000/uL	17,675	207.9 ± 96.1	214.4 ± 92.5	<0.001	208.8 ± 96.0	214.2 ± 92.5	<0.001	211.9 ± 99.0	212.8 ± 91.2	0.539
BUN, mg/dL	17,438	42.6 ± 31.8	29.3 ± 20.4	<0.001	41.9 ± 30.2	29.3 ± 21.2	<0.001	37.4 ± 29.0	31.3 ± 22.8	<0.001
Bicarbonate, mmol/L	8,916	22.1 ± 6.9	24.1 ± 6.3	<0.001	22.1 ± 6.7	24.1 ± 6.4	<0.001	22.7 ± 6.9	23.7 ± 6.4	<0.001
Sodium, mg/dL	17,665	137.1 ± 6.4	137.5 ± 5.6	<0.001	137.2 ± 6.3	137.5 ± 5.7	0.005	137.1 ± 6.3	137.5 ± 5.6	<0.001
Potassium, mg/dL	17,673	4.2 ± 0.8	4.0 ± 0.7	<0.001	4.2 ± 0.8	4.0 ± 0.7	<0.001	4.1 ± 0.8	4.0 ± 0.7	<0.001
Albumin, mg/dL	14,153	3.1 ± 0.7	3.4 ± 0.6	<0.001	3.1 ± 0.7	3.4 ± 0.6	<0.001	3.2 ± 0.7	3.4 ± 0.6	<0.001
Proteinuria, mg/dL	13,972			<0.001			<0.001			<0.001
Negative (0-4)		1,111 (25.9)	3,902 (40.3)		1,158 (25.9)	3,855 (40.6)		1,306 (29.5)	3,707 (38.8)	
Trace (5-29)		580 (13.5)	1,342 (13.8)		571 (12.8)	1,351 (14.2)		595 (13.5)	1,327 (13.9)	
≥1+ (≥30)		2,591 (60.5)	4,446 (45.9)		2,738 (61.3)	4,299 (45.2)		2,522 (57.0)	4,515 (47.3)	
BNP, pg/mL	8,275	1169 [529, 2321]	817 [385, 1570]	<0.001	1180 [559, 2360]	807 [381, 1561]	<0.001	1074 [492, 2180]	836 [395, 1603]	<0.001
NT-pro BNP, pg/mL	409	6488 [2860, 16984]	3096 [1257, 8927]	<0.001	7777 [3337, 16833]	3022 [1255, 7872]	<0.001	6379 [2608, 14476]	3179 [1255, 9005]	<0.001
Troponin-I, ng/ml	14,527	0.23 [0.05, 1.61]	0.09 [0.03, 0.50]	<0.001	0.23 [0.05, 1.50]	0.09 [0.04, 0.51]	<0.001	0.21 [0.05, 1.46]	0.09 [0.04, 0.53]	<0.001
Lactic acid, mg/dL	4,234	22.0 [12.9, 45.3]	18.0 [11.8, 30.0]	<0.001	21.1 [12.4, 43.3]	18.5 [12.2, 31.3]	<0.001	21.1 [12.6, 43.3]	18.4 [12.0, 31.5]	<0.001
pH	8,272	7.38 ± 0.12	7.40 ± 0.11	<0.001	7.38 ± 0.12	7.40 ± 0.11	<0.001	7.38 ± 0.12	7.40 ± 0.11	<0.001
Medication treatment										
ACEi/ARB	17,684	3,449 (68.3)	9,367 (74.1)	<0.001	3,652 (69.1)	9,164 (73.9)	<0.001	3,727 (70.5)	9,089 (73.3)	<0.001
Sacubitril/Valsartan	17,684	47 (0.9)	103 (0.8)	0.451	38 (0.7)	112 (0.9)	0.221	39 (0.7)	111 (0.9)	0.297

Variable	Available number	KIDGO definition AKI (*n* = 5,051)	KIDGO definition Non-AKI (*n* = 12,633)	*P*	WRF SCr definition AKI (*n* = 5,286)	WRF SCr definition Non-AKI (*n* = 12,398)	*P*	WRF GFR definition AKI (*n* = 5,284)	WRF GFR definition Non-AKI (*n* = 12,400)	*P*
Ivabradine	17,684	9 (0.2)	31 (0.2)	0.395	6 (0.1)	34 (0.3)	0.039	8 (0.2)	32 (0.3)	0.172
SGLT2i	17,684	52 (1.0)	194 (1.5)	0.009	41 (0.8)	205 (1.7)	<0.001	58 (1.1)	188 (1.5)	0.030
MRA	17,684	1,447 (28.6)	3,767 (29.8)	0.123	1,413 (26.7)	3,801 (30.7)	<0.001	1,516 (28.7)	3,698 (29.8)	0.131
Digoxin	17,684	1,246 (24.7)	2,751 (21.8)	<0.001	1,213 (22.9)	2,784 (22.5)	0.474	1,288 (24.4)	2,709 (21.8)	<0.001
Calcium channel blocker	17,684	2,978 (59.0)	5,977 (47.3)	<0.001	3,096 (58.6)	5,859 (47.3)	<0.001	3,046 (57.6)	5,909 (47.7)	<0.001
Beta-blocker	17,684	3,424 (67.8)	8,203 (64.9)	<0.001	3,570 (67.5)	8,057 (65.0)	0.001	3,546 (67.1)	8,081 (65.2)	0.013
Loop-diuretics	17,684	4,626 (91.6)	10,650 (84.3)	<0.001	4,837 (91.5)	10,439 (84.2)	<0.001	4,808 (91.0)	10,468 (84.4)	<0.001
Vasodilators	17,684	1,222 (24.2)	1,496 (11.8)	<0.001	1,330 (25.2)	1,388 (11.2)	<0.001	1,161 (22.0)	1,557 (12.6)	<0.001
NSAIDs	17,684	2,078 (41.1)	4,081 (32.3)	<0.001	2,057 (38.9)	4,102 (33.1)	<0.001	2,123 (40.2)	4,036 (32.5)	<0.001

Abbreviation: AKI, acute kidney injury; WRF, worsening renal fuction; SCr, serum creatinine; GFR, glomerular filtration rate; CKD, chronic kidney disease; eGFR, estimated glomerular filtration rate; LVEF, left ventricular ejection fraction; SBP, systolic blood pressure; DBP, diastolic blood pressure; BUN, blood urea nitrogen; BNP, B-type natriuretic peptide; ACEi, angiotensin converting enzyme inhibitor; ARB, angiotensin receptor blocker; SGLT2i, sodium-glucose cotransporter 2 inhibitor; MRA, mineralocorticoid receptor antagonist NSAIDs, non-steroidal anti-inflammatory drugs;

Data were presented as frequency (percentage), mean ± standard deviation or median [25th, 75th percentiles].

Supplementary Table 2. Agreement of AKI stage between KDIGO and WRF SCr and WRF GFR classifications

	WRF SCr					
KDIGO	Non-AKI	1.5x	2x	3x	Dialysis	Total
Non-AKI	11,588 (65.53)	1,017 (5.75)	21 (0.12)	7 (0.04)	0 (0.0)	12,633 (71.44)
Stage 1: 1.5x	691 (3.91)	1,707 (9.65)	155 (0.88)	62 (0.35)	0 (0.0)	2,615 (14.79)
Stage 2: 2x	62 (0.35)	99 (0.56)	471 (2.66)	55 (0.31)	0 (0.0)	687 (3.88)
Stage 3: 3x	57 (0.32)	409 (2.31)	24 (0.14)	234 (1.32)	0 (0.0)	724 (4.09)
Dialysis	0 (0.0)	0 (0.0)	0 (0.0)	0 (0.0)	1,025 (5.80)	1,025 (5.80)
Total	12,398 (70.11)	3,232 (18.28)	671 (3.79)	358 (2.02)	1,025 (5.80)	17,684 (100)
	WRF GFR					
KDIGO	Non-AKI	20-50	51-75	>75	Dialysis	Total
Non-AKI	11,308 (63.94)	1,288 (7.28)	32 (0.18)	5 (0.03)	0 (0.0)	12,633 (71.44)
Stage 1: 1.5x	760 (4.30)	1,433 (8.10)	381 (2.15)	41 (0.23)	0 (0.0)	2,615 (14.79)
Stage 2: 2x	53 (0.30)	90 (0.51)	500 (2.83)	44 (0.25)	0 (0.0)	687 (3.88)
Stage 3: 3x	279 (1.58)	172 (0.97)	84 (0.48)	189 (1.07)	0 (0.0)	724 (4.09)
Dialysis	0 (0.0)	0 (0.0)	0 (0.0)	0 (0.0)	1,025 (5.80)	1,025 (5.80)
Total	12,400 (70.12)	2,983 (16.87)	997 (5.64)	279 (1.58)	1,025 (5.80)	17,684 (100)

Abbreviation: AKI, acute kidney injury; WRF, worsening renal function; SCr, serum creatinine; KDIGO, Kidney Disease: Improving Global Outcomes; GFR, glomerular filtration rate;

Data were presented as frequency (percentage).

Supplementary Table 3. The performance of AKI stage under different AKI/WRF classifications to discriminate outcomes

	AUC (95% CI)			
Outcome / Stage classifications	KIDGO stage	WRF SCr stage	WRF GFR stage	Modified KIDGO‡
In-hospital death	74.8 (73.8–75.8)	74.0 (72.9–75.0)	73.7 (72.6–74.7)	75.2 (74.2–76.2)
3 months follow-up				
All-cause death	53.4 (52.1–54.7)	54.0 (52.6–55.3)	53.0 (51.6–54.3)	53.3 (52.0–54.6)
Heart failure hospitalization	50.9 (49.3–52.4)	52.0 (50.4–53.7)	50.3 (48.7–51.8)	50.8 (49.3–52.3)
1 year follow-up				
MAKE#	51.5 (50.5–52.6)	51.4 (50.5–52.4)	50.6 (49.5–51.7)	51.6 (50.5–52.6)
All-cause death	52.4 (51.6–53.3)	52.8 (51.9–53.7)	52.1 (51.2–52.9)	52.3 (51.5–53.2)
Heart failure hospitalization	50.3 (49.1–51.4)	49.3 (48.1–50.5)	50.8 (49.7–52.0)	50.3 (49.2–51.4)

Abbreviation: AKI, acute kidney injury; AUC, area under the curve; CI, confidence interval; KDIGO, Kidney Disease: Improving Global Outcomes; SCr, serum creatinine; GFR, glomerular filtration rate; MAKE, major adverse kidney event;

‡ Increase in serum creatinine to ≥4.0 mg/dl and ≥0.3 mg/dl increase changed to stage 1;

# MAKE within 3-months AKI recovery period post-hospital discharge was not used as event identification.

Supplementary Table 4. Outcomes during 1-year follow up after discharge under different AKI/WRF classifications

	AKI or WRF = yes		AKI or WRF = no			
Outcome / Definition	*n*	Event (%)	*n*	Event (%)	AUC, % (95% CI)	aOR (95% CI)†
MAKE‡						
KIDGO	698	267 (38.3)	4,329	1411 (32.6)	51.5 (50.5–52.6)	**1.41 (1.18–1.68)***
WRF SCr	548	214 (39.1)	4,479	1464 (32.7)	51.4 (50.4–52.3)	**1.52 (1.25–1.84)***
WRF GFR	800	278 (34.8)	4,227	1400 (33.1)	50.5 (49.4–51.6)	**1.37 (1.16–1.63)***
All-cause death						
KIDGO	3,317	818 (24.7)	11,854	2,385 (20.1)	52.3 (51.5–53.2)	**1.37 (1.24–1.51)***
WRF SCr	3,604	901 (25.0)	11,567	2,302 (19.9)	52.8 (51.9–53.6)	**1.33 (1.22–1.46)***
WRF GFR	3,620	865 (23.9)	11,551	2,338 (20.2)	52.0 (51.1–52.9)	**1.32 (1.20–1.44)***
Heart failure hospitalization						
KIDGO	3,317	297 (9.0)	11,854	1,077 (9.1)	50.1 (49.0–51.3)	0.90 (0.78–1.03)
WRF SCr	3,604	351 (9.7)	11,567	1,023 (8.8)	51.0 (49.8–52.2)	0.99 (0.87–1.13)
WRF GFR	3,620	311 (8.6)	11,551	1,063 (9.2)	50.7 (49.5–51.8)	0.91 (0.80–1.04)

Abbreviation: AKI, acute kidney injury; WRF, worsening renal function; AUC, area under the curve; aOR, adjusted odds ratio; CI, confidence interval; KDIGO, Kidney Disease: Improving Global Outcomes; SCr, serum creatinine; GFR, glomerular filtration rate;

† Adjusted for eGFR at admission, age, gender, underlying diabetes mellitus, dyslipidemia, hypertension, myocardial infarction and atrial fibrillation;

‡ Only patients with available serum creatinine data after 3 months of discharge (AKI recovery period) were analyzed.

Supplementary Table 5. Outcomes during 1-year follow up after discharge by different AKI stages under different classifications

	KIDGO			WRF SCr			WRF GFR		
Outcome / Stage	*n*	Event (%)	aOR (95% CI)†	*n*	Event (%)	aOR (95% CI)†	*n*	Event (%)	aOR (95% CI)†
MAKE‡									
0	4,329	1,411 (32.6)	Reference	4,479	1,464 (32.7)	Reference	4,227	1,400 (33.1)	Reference
1	546	206 (37.7)	**1.39 (1.14–1.68)***	420	160 (38.1)	**1.48 (1.20–1.84)***	628	208 (33.1)	**1.31 (1.08–1.58)***
2	100	38 (38.0)	1.37 (0.89–2.10)	75	29 (38.7)	1.35 (0.83–2.20)	130	49 (37.7)	1.40 (0.96–2.06)
3	34	12 (35.3)	1.31 (0.62–2.76)	35	14 (40.0)	1.82 (0.88–3.77)	24	10 (41.7)	2.03 (0.86–4.82)
Dialysis	18	11 (61.1)	**2.98 (1.11–8.00)***	18	11 (61.1)	**2.97 (1.11–7.95)***	18	11 (61.1)	**2.95 (1.10–7.91)***
All-cause death									
0	11,854	2,385 (20.1)	Reference	11,567	2,302 (19.9)	Reference	11,551	2,338 (20.2)	Reference
1	1,908	457 (24.0)	**1.32 (1.17–1.48)***	2,560	636 (24.8)	**1.29 (1.16–1.44)***	2,408	562 (23.3)	**1.27 (1.14–1.41)***
2	371	83 (22.4)	1.19 (0.92–1.54)	340	83 (24.4)	1.29 (0.99–1.67)	545	130 (23.9)	**1.31 (1.06–1.61)***
3	468	128 (27.4)	**1.56 (1.25–1.95)***	134	32 (23.9)	1.52 (0.99–2.31)	97	23 (23.7)	1.58 (0.97–2.60)*
Dialysis	570	150 (26.3)	**1.56 (1.27–1.92)***	570	150 (26.3)	**1.55 (1.26–1.91)***	570	150 (26.3)	**1.50 (1.22–1.84)***
HHF									
0	11,854	1,077 (9.1)	Reference	11,567	1,023 (8.8)	Reference	11,551	1,063 (9.2)	Reference
1	1,908	183 (9.6)	1.06 (0.90–1.25)	2,560	277 (10.8)	1.13 (0.98–1.30)	2,408	224 (9.3)	1.05 (0.90–1.23)
2	371	24 (6.5)	0.72 (0.47–1.10)	340	22 (6.5)	0.72 (0.46–1.11)	545	38 (7.0)	0.78 (0.55–1.09)
3	468	52 (11.1)	0.95 (0.70–1.29)	134	14 (10.5)	1.34 (0.77–2.36)	97	11 (11.3)	1.51 (0.80–2.86)
Dialysis	570	38 (6.7)	**0.52 (0.37–0.73)***	570	38 (6.7)	**0.53 (0.38–0.76)***	570	38 (6.7)	**0.52 (0.37–0.73)***

Abbreviation: AKI, acute kidney injury; KDIGO, Kidney Disease: Improving Global Outcomes; WRF, worsening renal function; SCr, serum creatinine; GFR, glomerular filtration rate; aOR, adjusted odds ratio; CI, confidence interval; MAKE, major adverse kidney event; HHF, heart failure hospitalization;

† Adjusted for eGFR at admission, age, gender, underlying diabetes mellitus, dyslipidemia, hypertension, myocardial infarction and atrial fibrillation;

‡ Only patients with available serum creatinine data after 3 months of discharge (AKI recovery period) were analyzed.

Supplementary Table 6. Baseline characteristics of patients with WRF (either GFR or SCr definitions) who fell outside of the KDIGO-AKI time window and who did not

Variable	WRF (+) and KDIGO (+) (*n* = 4,342)	WRF (+) and KDIGO (-) (*n* = 1,541)	*P* value
Baseline demographics			
Age, year	70.9 ± 14.6	72.5 ± 14.2	<0.001
Male	2,431 (56.0)	788 (51.1)	0.001
Body mass index, kg/m^2	24.6 ± 5.0	24.9 ± 5.3	0.099
Renal function at admission			
Creatinine, mg/dL	2.6 ± 2.6	1.4 ± 0.8	<0.001
eGFR, mL/min/1.73m^2	48.3 ± 43.0	64.9 ± 40.3	<0.001
eGFR stages			<0.001
G1 (≥90)	499 (11.5)	335 (21.7)	
G2 (60-89)	828 (19.1)	412 (26.7)	
G3a (45-59)	661 (15.2)	228 (14.8)	
G3b (30-44)	693 (16.0)	235 (15.2)	
G4 (15-29)	728 (16.8)	288 (18.7)	
G5 (<15)	933 (21.5)	43 (2.8)	
Comorbidity			
Diabetes mellitus	2,046 (47.1)	733 (47.6)	0.764
Dyslipidemia	1,366 (31.5)	557 (36.1)	0.001
Hypertension	2,899 (66.8)	1,073 (69.6)	0.039
Myocardial infarction	248 (5.7)	101 (6.6)	0.229

Variable	WRF (+) and KDIGO (+) (*n* = 4,342)	WRF (+) and KDIGO (-) (*n* = 1,541)	*P* value
Atrial fibrillation	1,140 (26.3)	442 (28.7)	0.065
Heart function			
LVEF, %	62.8 ± 14.0	63.4 ± 13.4	0.377
LVEF group			0.764
<40% (Reduced)	98 (7.7)	34 (7.0)	
40-54%	177 (13.9)	63 (13.0)	
≥55% (Preserved)	994 (78.3)	386 (79.9)	
Vital sign			
SBP, mmHg	135.3 ± 30.4	139.6 ± 27.9	<0.001
DBP, mmHg	76.1 ± 18.8	78.1 ± 18.0	0.001
Heart rate, beat/min	92.5 ± 22.8	89.8 ± 21.4	<0.001
Baseline lab data			
Hemoglobin, g/dL	11.1 ± 2.6	11.5 ± 2.5	<0.001
Platelets, 1000/uL	207.2 ± 95.9	221.5 ± 103.7	<0.001
BUN, mg/dL	43.2 ± 31.9	28.9 ± 18.6	<0.001
Bicarbonate, mmol/L	21.9 ± 6.8	24.4 ± 6.5	<0.001
Sodium, mg/dL	137.2 ± 6.4	137.1 ± 5.9	0.512
Potassium, mg/dL	4.2 ± 0.8	4.0 ± 0.7	<0.001
Albumin, mg/dL	3.1 ± 0.7	3.3 ± 0.6	<0.001
Proteinuria, mg/dL			<0.001
Negative (0-4)	928 (25.2)	449 (35.6)	

Variable	WRF (+) and KDIGO (+) (*n* = 4,342)	WRF (+) and KDIGO (-) (*n* = 1,541)	*P* value
Trace (5-29)	482 (13.1)	162 (12.8)	
≥1+ (≥30)	2,267 (61.7)	652 (51.6)	
BNP, pg/mL	1205 [554, 2380]	881 [416, 1767]	<0.001
NT-pro BNP, pg/mL	7592 [3253, 17260]	3492 [1633, 13743]	0.094
Troponin-I, ng/ml	0.24 [0.06, 1.77]	0.11 [0.04, 0.72]	<0.001
Lactic acid, mg/dL	21.8 [12.7, 46.4]	17.4 [11.2, 27.0]	<0.001
pH	7.37 ± 0.12	7.40 ± 0.10	<0.001
Medication treatment			
ACEi/ARB	2,920 (67.3)	1,205 (78.2)	<0.001
Sacubitril/Valsartan	35 (0.81)	6 (0.39)	0.091
Ivabradine	6 (0.14)	2 (0.13)	0.939
SGLT2i	37 (0.85)	22 (1.43)	0.051
MRA	1,158 (26.7)	472 (30.6)	0.003
Digoxin	1,009 (23.2)	363 (23.6)	0.800
Calcium channel blocker	2,569 (59.2)	859 (55.7)	0.019
Beta-blocker	2,927 (67.4)	1,053 (68.3)	0.507
Loop-diuretics	3,964 (91.3)	1,406 (91.2)	0.948
Vasodilators	1,100 (25.3)	271 (17.6)	<0.001
NSAIDs	1,752 (40.4)	539 (35.0)	<0.001

Abbreviation: KDIGO, Kidney Disease: Improving Global Outcomes; CKD, chronic kidney disease; eGFR, estimated glomerular filtration rate; LVEF, left ventricular ejection fraction; SBP, systolic blood pressure; DBP, diastolic blood pressure; BUN, blood urea nitrogen; BNP, B-type

natriuretic peptide; ACEi, angiotensin converting enzyme inhibitor; ARB, angiotensin receptor blocker; SGLT2i, sodium-glucose cotransporter 2 inhibitor; MRA, mineralocorticoid receptor antagonist NSAIDs, non-steroidal anti-inflammatory drugs;

Data were presented as frequency (percentage), mean ± standard deviation or median [25th, 75th percentiles].

Supplementary Table 7. Outcomes after discharge of patients with WRF (either GFR or SCr definitions) who fell outside of the KDIGO-AKI time window and who did not

Outcome / Definition	WRF (+) and KDIGO (+) *n*	WRF (+) and KDIGO (+) Event (%)	WRF (+) and KDIGO (-) *n*	WRF (+) and KDIGO (-) Event (%)	aOR (95% CI) of WRF (+) and KDIGO (-)§
In-hospital death	4,342	1,584 (36.5)	1,541	164 (10.6)	**0.19 (0.16–0.22)***
3-month follow up					
All-cause death†	2,758	296 (10.7)	1,377	136 (9.9)	0.82 (0.66–1.03)
Heart failure hospitalization†	2,758	154 (5.6)	1,377	73 (5.3)	0.98 (0.73–1.33)
1-year follow up					
MAKE‡	485	188 (38.8)	327	97 (29.7)	**0.68 (0.50–0.94)***
All-cause death†	2,758	676 (24.5)	1,377	318 (23.1)	0.87 (0.74–1.02)
Heart failure hospitalization†	2,758	257 (9.3)	1,377	127 (9.2)	1.04 (0.82–1.32)

Abbreviation: KDIGO, Kidney Disease: Improving Global Outcomes; aOR, adjusted odds ratio; CI, confidence interval;

§ Adjusted for eGFR at admission, age, gender, underlying diabetes mellitus, dyslipidemia, hypertension, myocardial infarction and atrial fibrillation;

† Only patients survived after discharge were analyzed;

‡ Only patients with available serum creatinine data after 3 months of discharge (AKI recovery period) were analyzed.
